# Supplementary material for: Underweight, overweight or obesity, diabetes, and hypertension in Bangladesh, 2004 to 2018
Source: PLoS One. 2022 Sep 30;17(9):e0275151. doi: 10.1371/journal.pone.0275151 (PMC9524627; doi:10.1371/journal.pone.0275151)
Supplement: S1 Fig — (DOCX) [file pone.0275151.s001.docx]

**S1 Figure: Prevalance of underweight and overweight/obesity among women and men by age group, Bangladesh 2004 – 2018**

| 1. **Underweight** |
| --- |
|  |
| 1. **Overweight/obesity** |
|  |
