# Supplementary material for: Underweight, overweight or obesity, diabetes, and hypertension in Bangladesh, 2004 to 2018
Source: PLoS One. 2022 Sep 30;17(9):e0275151. doi: 10.1371/journal.pone.0275151 (PMC9524627; doi:10.1371/journal.pone.0275151)
Supplement: S1 Table — The following methods are adapted from UNICEF (42). When prevalence estimates are available for multiple years in a country, the AARC can be calculated using a regression analysis as follows: If the prevalence in a baseline year t0 is Y0 and four data points after t0 are available for trend analysis, then each of the four points can be written as: Yti = Y0*(1-b%) (ti -t0), so that, ln(Yti) = ln(Y0) + (ti -t0)*ln(1-b%) = ln(Y0) + ti *ln(1-b%)–t0*ln(1-b%) = β*ti + C0, Where β = ln(1-b%) and C0 = ln(Y0)–t0*ln(1+b%), a constant β, the coefficient of ti, in a simple linear regression of ln(Yi) against ti can then be translated into b%. The AARC = (EXP(β) *100)- 100. (DOCX) [file pone.0275151.s002.docx]

**S1 Table. Estimating average annual rate of change (AARC) in underweight, overweight/obesity and noncommunicable diseases in women (2004-2018) and men (2011-2018)**

|  | **Women** | | | **Men** | | |
| --- | --- | --- | --- | --- | --- | --- |
|  | Year | Prevalence  (%) | Log of  prevalence | Year | Prevalence  (%) | Log of  prevalence |
| **Underweight** | 2004 | 33.1 | 3.50 |  |  |  |
|  | 2007 | 28.7 | 3.36 |  |  |  |
|  | 2011 | 24.2 | 3.19 | 2011 | 28.2 | 3.34 |
|  | 2014 | 18.3 | 2.91 |  |  |  |
|  | 2018 | 12.2 | 2.50 | 2018 | 19.8 | 2.99 |
| Slope | -0.07 | | | -0.05 | | |
| AARC | -6.77 | | | -4.93 | | |
| **Overweight** | 2004 | 17.3 | 2.85 |  |  |  |
|  | 2007 | 21.8 | 3.08 |  |  |  |
|  | 2011 | 28.9 | 3.36 | 2011 | 19.8 | 2.99 |
|  | 2014 | 38.8 | 3.66 |  |  |  |
|  | 2018 | 48.5 | 3.88 | 2018 | 32.6 | 3.48 |
| Slope | 0.08 | | | 0.07 | | |
| AARC | 7.81 | | | 7.38 | | |
| **Diabetes** |  | | |  | | |
|  | 2011 | 11.3 | 2.42 | 2011 | 10.7 | 2.37 |
|  | 2018 | 14.1 | 2.65 | 2018 | 14.1 | 2.65 |
| Slope | 0.03 | | | 0.04 | | |
| AARC | 3.21 | | | 4.02 | | |
| **Hypertension** |  | | |  | | |
|  | 2011 | 31.1 | 3.44 | 2011 | 18.6 | 2.92 |
|  | 2018 | 43.8 | 3.78 | 2018 | 33.2 | 3.50 |
| Slope | 0.05 | | | 0.08 | | |
| AARC | 5.01 | | | 8.63 | | |

The following methods are adapted from UNICEF [42]. When prevalence estimates are available for multiple years in a country, the AARC can be calculated using a regression analysis as follows:

Y_ti_ = Y_0_*(1+b%)^(ti -t0)^,

Where Y_0_ and Y_ti_ are the prevalence at baseline and time t_i_, respectively. b% is the AARC

ln(Y_ti_) = ln(Y_0_) + (ti -t_0_)*ln(1+b%) = ln(Y_0_) + t_i_ *ln(1+b%) – t_0_*ln(1+b%) = β*t_i_ + C_0_

Where β = ln(1+b%) and C_0_ = ln(Y_0_) – t_0_*ln(1+b%) is a constant; β is the coefficient of t_i_, in a simple linear regression of ln(Y_i_ ) against t_i_ can then be translated into b%.

The AARC= (EXP(β) *100) - 100
