# Supplementary material for: Underweight, overweight or obesity, diabetes, and hypertension in Bangladesh, 2004 to 2018
Source: PLoS One. 2022 Sep 30;17(9):e0275151. doi: 10.1371/journal.pone.0275151 (PMC9524627; doi:10.1371/journal.pone.0275151)
Supplement: S2 Table — 1Negative values mean that the burden is more concentrated in the poor and positive values mean that the burden is more concentrated in the wealthy. *,**,*** Significant difference for inequality between Q1 and Q5: *P<0.05, **P<0.01, *** P < 0.001; Q: quintile. SII: Slope Index of Inequality, CIX: Concentration Index. (DOCX) [file pone.0275151.s003.docx]

**S2 Table. Socio-economic inequality in underweight, overweight/obesity, and noncommunicable diseases among women and men by survey round, Bangladesh 2004 – 2018**

|  | **Year** | **Q1** | **Q5** | **SII^1^** | **CIX** |
| --- | --- | --- | --- | --- | --- |
| **Women** |  |  |  |  |  |
| Underweight | 2004 | 45.9 | 17.0 | -36.41^***^ | -5.40^***^ |
|  | 2007 | 41.5 | 13.4 | -34.23^***^ | -5.22^***^ |
|  | 2011 | 39.7 | 8.5 | -35.98^***^ | -5.78^***^ |
|  | 2014 | 31.1 | 7.0 | -28.91^***^ | -4.78^***^ |
|  | 2018 | 20.6 | 5.1 | -19.36^***^ | -3.01^***^ |
| Overweight/obesity | 2004 | 6.0 | 38.9 | 44.34^***^ | 6.36^***^ |
|  | 2007 | 8.3 | 48.1 | 51.42^***^ | 7.55^***^ |
|  | 2011 | 11.1 | 55.1 | 56.03^***^ | 8.61^***^ |
|  | 2014 | 18.2 | 64.3 | 55.30^***^ | 8.97^***^ |
|  | 2018 | 30.8 | 69.9 | 47.98^***^ | 7.56^***^ |
| Diabetes | 2011 | 6.7 | 21.5 | 18.42^***^ | 2.84^***^ |
|  | 2018 | 8.7 | 26.7 | 22.85^***^ | 3.52^***^ |
| Hypertension | 2011 | 24.2 | 42.8 | 22.09^***^ | 3.51^***^ |
|  | 2018 | 40.3 | 49.8 | 13.93^***^ | 2.05^***^ |
| **Men** |  |  |  |  |  |
| Underweight | 2011 | 41.2 | 12.1 | -36.63^***^ | -5.74^***^ |
|  | 2018 | 30.7 | 8.2 | -25.96^***^ | -4.41^***^ |
| Overweight/obesity | 2011 | 4.4 | 45.1 | 52.16^***^ | 7.87^***^ |
|  | 2018 | 15.5 | 56.5 | 50.07^***^ | 8.05^***^ |
| Diabetes | 2011 | 7.9 | 19.5 | 16.07^***^ | 2.14^***^ |
|  | 2018 | 5.8 | 30.2 | 25.36^***^ | 4.58^***^ |
| Hypertension | 2011 | 12.2 | 29.6 | 22.96^***^ | 3.26^***^ |
|  | 2018 | 25.7 | 45.0 | 25.36^***^ | 3.58^***^ |

**^1^**Negative values mean that the burden is more concentrated in the poor and positive values mean that the burden is more concentrated in the wealthy. *,**,*** Significant difference for inequality between Q1 and Q5: **P<0.05,* **P<0.01, *** *P* < 0.001; Q: quintile. SII: Slope Index of Inequality, CIX: Concentration Index
