# Supplementary material for: Underweight, overweight or obesity, diabetes, and hypertension in Bangladesh, 2004 to 2018
Source: PLoS One. 2022 Sep 30;17(9):e0275151. doi: 10.1371/journal.pone.0275151 (PMC9524627; doi:10.1371/journal.pone.0275151)
Supplement: S3 Table — 1Negative values mean that the burden is more concentrated in the rural and positive values mean that the burden is more concentrated in the urban. *,**,*** Significant difference for inequality between rural and urban areas: *P<0.05, **P<0.01, *** P < 0.001; SII: Slope Index of Inequality, CIX: Concentration Index. (DOCX) [file pone.0275151.s004.docx]

**S3 Table. Residential inequality in underweight, overweight/obesity, and noncommunicable diseases among women and men by survey round, Bangladesh 2004 – 2018**

|  | **Year** | **Rural** | **Urban** | **SII^1^** | **CIX** |
| --- | --- | --- | --- | --- | --- |
| **Women** |  |  |  |  |  |
| Underweight | 2004 | 35.7 | 24.5 | -23.92^***^ | -1.93^***^ |
|  | 2007 | 31.4 | 19.2 | -23.97^***^ | -2.12^***^ |
|  | 2011 | 27.8 | 13.7 | -25.01^***^ | -2.67^***^ |
|  | 2014 | 20.7 | 12.1 | -17.31^***^ | -1.73^***^ |
|  | 2018 | 13.4 | 9.0 | -9.74^***^ | -0.88^***^ |
| Overweight | 2004 | 13.5 | 30.6 | 36.94^***^ | 2.97^***^ |
|  | 2007 | 16.9 | 38.9 | 40.00^***^ | 3.82^***^ |
|  | 2011 | 23.5 | 45.0 | 42.34^***^ | 4.08^***^ |
|  | 2014 | 33.4 | 52.6 | 38.92^***^ | 3.87^***^ |
|  | 2018 | 44.4 | 59.2 | 30.06^***^ | 2.98^***^ |
| Diabetes | 2011 | 9.5 | 11.8 | 14.21^***^ | 1.37^***^ |
|  | 2018 | 17.3 | 21.7 | 18.8^***^ | 1.79^***^ |
| Hypertension | 2011 | 28.8 | 42.7 | 18.78^***^ | 1.82^***^ |
|  | 2018 | 39.1 | 47.5 | 8.11^***^ | 0.89^*^ |
| **Men** |  |  |  |  |  |
| Underweight | 2011 | 31.4 | 18.7 | -20.90^***^ | -2.39^***^ |
|  | 2018 | 22.0 | 14.2 | -14.71^***^ | -1.57^***^ |
| Overweight | 2011 | 15.2 | 33.4 | 34.53^***^ | 3.40^***^ |
|  | 2018 | 28.9 | 42.1 | 27.54^***^ | 2.68^***^ |
| Diabetes | 2011 | 9.3 | 12.8 | 11.83^***^ | 1.02^***^ |
|  | 2018 | 14.9 | 17.9 | 7.97^**^ | 0.98^***^ |
| Hypertension | 2011 | 16.9 | 32.0 | 15.92^***^ | 1.30^***^ |
|  | 2018 | 24.2 | 36.7 | 10.69^**^ | 0.89^*^ |

**^1^**Negative values mean that the burden is more concentrated in the rural and positive values mean that the burden is more concentrated in the urban. *,**,*** Significant difference for inequality between rural and urban areas: **P<0.05,* **P<0.01, *** *P* < 0.001; SII: Slope Index of Inequality, CIX: Concentration Index
