# Supplementary material for: Underweight, overweight or obesity, diabetes, and hypertension in Bangladesh, 2004 to 2018
Source: PLoS One. 2022 Sep 30;17(9):e0275151. doi: 10.1371/journal.pone.0275151 (PMC9524627; doi:10.1371/journal.pone.0275151)
Supplement: S4 Table — (DOCX) [file pone.0275151.s005.docx]

**S4 Table. Number and percentage of individuals with the outcomes, by selected factors**

|  | **Underweight** | **Overweight/obesity** | **Diabetes** | **Hypertension** |
| --- | --- | --- | --- | --- |
|  | **n (%)** | **n (%)** | **n (%)** | **n (%)** |
| **Women** |  |  |  |  |
| **Household SES** |  |  |  |  |
| Quintile 1 | 4623 (33.9) | 2335 (17.1) | 109 (7.8) | 476 (32.8) |
| Quintile 2 | 3921 (27.6) | 3108 (21.9) | 109 (8.1) | 488 (34.2) |
| Quintile 3 | 3376 (22.6) | 4480 (30.0) | 141 (9.7) | 539 (35.8) |
| Quintile 4 | 2836 (17.9) | 6164 (38.8) | 198 (13.4) | 602 (39.1) |
| Quintile 5 | 1704 (9.5) | 10486 (58.2) | 418 (24.9) | 840 (47.0) |
| **Exposed to media** |  |  |  |  |
| No | 10764 (27.8) | 9094 (23.5) | 356 (9.4) | 1380 (34.9) |
| Yes | 5695 (15.0) | 17479 (46.0) | 619 (17.3) | 1565 (41.7) |
| **Place of residence** |  |  |  |  |
| Urban | 4162 (15.3) | 12728 (46.8) | 451 (18.7) | 1091 (42.8) |
| Rural | 12298 (24.9) | 13845 (28.0) | 524 (10.6) | 1854 (35.9) |
| **Improved latrine** |  |  |  |  |
| No | 8315 (28.4) | 6969 (23.8) | 295 (10.4) | 1015 (34.0) |
| Yes | 8144 (17.2) | 19602 (41.4) | 680 (15.1) | 1930 (40.8) |
| **Improved drinking water** |  |  |  |  |
| No | 636 (29.0) | 543 (24.7) | 19 (10.7) | 70 (38.5) |
| Yes | 15823 (21.2) | 26028 (35.0) | 956 (13.3) | 2875 (38.2) |
| **Individual level** |  |  |  |  |
| **Education** |  |  |  |  |
| No education | 5919 (29.9) | 4595 (23.2) | 393 (10.4) | 1535 (38.9) |
| ≤ Primary school | 5308 (23.1) | 7187 (31.3) | 298 (13.7) | 828 (36.3) |
| ≤ Secondary school | 4546 (17.2) | 10573 (40.0) | 207 (19.5) | 446 (40.4) |
| College or higher | 686 (9.2) | 4216 (56.6) | 77 (22.3) | 136 (36.5) |
| **Currently working** |  |  |  |  |
| No | 11732 (22.1) | 18232 (34.3) | 758 (14.2) | 2214 (39.6) |
| Yes | 4718 (20.0) | 8332 (35.4) | 217 (10.7) | 730 (34.6) |
| **Having children <5y** |  |  |  |  |
| No | 6979 (18.7) | 14777 (39.6) | 653 (13.7) | 1923 (38.5) |
| Yes | 9481 (24.1) | 11796 (30.0) | 322 (12.4) | 1022 (37.6) |
| **Overweight/obesity** |  |  |  |  |
| No | -- | -- | 385 (8.9) | 1371 (30.4) |
| Yes | -- | -- | 551 (19.6) | 1462 (49.3) |
| **Men** |  | |  | |
| **Household SES** |  |  |  |  |
| Quintile 1 | 692 (34.5) | 207 (10.3) | 91 (6.8) | 262 (18.7) |
| Quintile 2 | 641 (31.2) | 311 (15.1) | 93 (7.0) | 299 (21.1) |
| Quintile 3 | 542 (25.3) | 470 (22.0) | 117 (8.6) | 346 (23.9) |
| Quintile 4 | 411 (18.6) | 693 (31.4) | 176 (12.7) | 407 (28.2) |
| Quintile 5 | 247 (9.9) | 1305 (52.3) | 386 (23.5) | 645 (37.7) |
| **Exposed to media** |  |  |  |  |
| No | 1616 (29.6) | 941 (17.2) | 298 (8.4) | 847 (22.6) |
| Yes | 917 (16.9) | 2045 (37.6) | 565 (16.1) | 1112 (30.3) |
| **Place of residence** |  |  |  |  |
| Urban | 675 (17.3) | 1469 (37.7) | 376 (15.6) | 786 (31.2) |
| Rural | 1858 (26.5) | 1517 (21.7) | 487 (10.5) | 1173 (24.0) |
| **Improved latrine** |  |  |  |  |
| No | 1224 (29.0) | 720 (17.0) | 241 (8.9) | 597 (20.8) |
| Yes | 1309 (19.6) | 2266 (34.0) | 622 (14.3) | 1362 (29.9) |
| **Improved drinking water** |  |  |  |  |
| No | 50 (22.9) | 51 (23.4) | 14 (9.4) | 48 (30.6) |
| Yes | 2483 (23.2) | 2935 (27.5) | 849 (12.3) | 1911 (26.3) |
| **Individual level** |  |  |  |  |
| **Education** |  |  |  |  |
| No education | 985 (34.4) | 374 (13.1) | 183 (7.9) | 511 (20.7) |
| ≤ Primary school | 862 (25.6) | 711 (21.1) | 235 (11.2) | 541 (24.5) |
| ≤ Secondary school | 530 (18.5) | 938 (32.7) | 235 (14.1) | 518 (30.0) |
| College or higher | 156 (8.7) | 962 (53.4) | 209 (21.5) | 389 (38.6) |
| **Manual worker** |  |  |  |  |
| No | 878 (19.0) | 1724 (37.4) | 551 (16.0) | 1189 (33.1) |
| Yes | 1263 (26.3) | 981 (20.4) | 304 (8.5) | 760 (20.1) |
| **Overweight/obesity** |  |  |  |  |
| No | -- | -- | 455 (9.2) | 1064 (20.5) |
| Yes | -- | -- | 385 (19.5) | 840 (40.4) |
