# Supplementary material for: Underweight, overweight or obesity, diabetes, and hypertension in Bangladesh, 2004 to 2018
Source: PLoS One. 2022 Sep 30;17(9):e0275151. doi: 10.1371/journal.pone.0275151 (PMC9524627; doi:10.1371/journal.pone.0275151)
Supplement: S5 Table — Source: FAOstat website: http://www.fao.org/faostat/en/#data/FBS. (DOCX) [file pone.0275151.s006.docx]

**S5 Table. Supply of different food groups Bangladesh between 2010-2019 (kg/capita/year)**

|  | **2010** | **2011** | **2012** | **2013** | **2014** | **2015** | **2016** | **2017** | **2018** | **2019** | **% change**  **between 2010-2019** |
| --- | --- | --- | --- | --- | --- | --- | --- | --- | --- | --- | --- |
| Cereals | 275.51 | 279.92 | 280.13 | 275.85 | 275.24 | 281.35 | 270.66 | 277.86 | 287.13 | 288.35 | 4.7 |
| Roots | 47.37 | 47.83 | 48.53 | 49.25 | 49.91 | 50.7 | 51.7 | 52.37 | 53.27 | 54.22 | 14.5 |
| Pulses | 5.24 | 4.82 | 4.94 | 5.67 | 5.05 | 5.3 | 6.34 | 6.18 | 6.9 | 7.13 | 36.1 |
| Vegetables | 26.78 | 26.62 | 28.11 | 28.22 | 30.6 | 34.48 | 33.93 | 36.46 | 36.46 | 39.02 | 45.7 |
| Fruits | 24.93 | 24.38 | 23.95 | 24.34 | 24.59 | 27 | 27.46 | 28.78 | 27.77 | 28.76 | 15.4 |
| Meat | 3.91 | 3.89 | 3.97 | 4.1 | 4.37 | 4.42 | 4.47 | 4.27 | 4.23 | 4.24 | 8.4 |
| Eggs | 1.51 | 1.58 | 1.87 | 1.93 | 2.5 | 2.67 | 2.86 | 3.6 | 3.65 | 2.78 | 84.1 |
| Milk | 18.48 | 18.84 | 18.68 | 18.5 | 18.41 | 18.16 | 17.82 | 17.68 | 17.51 | 17.53 | -5.1 |
| Fish and sea foods | 19.99 | 20.27 | 21.24 | 22.1 | 22.92 | 23.86 | 24.5 | 26.01 | 25.73 | 25.47 | 27.4 |
| Vegetable oils | 5.07 | 5.21 | 5.43 | 5.95 | 6.72 | 7.12 | 6.91 | 7.03 | 7.2 | 7.2 | 42.0 |
| Sugar & sweeteners | 10.9 | 10.96 | 10.98 | 11.11 | 10.83 | 10.88 | 10.69 | 10.28 | 10.25 | 10.18 | -6.6 |
| Alcoholic beverages | 0.04 | 0.02 | 0.02 | 0.02 | 0.02 | 0.02 | 0.02 | 0.02 | 0.03 | 0.03 | -25.0 |

Source: FAOstat website: http://www.fao.org/faostat/en/#data/FBS
