# Supplementary material for: Underweight, overweight or obesity, diabetes, and hypertension in Bangladesh, 2004 to 2018
Source: PLoS One. 2022 Sep 30;17(9):e0275151. doi: 10.1371/journal.pone.0275151 (PMC9524627; doi:10.1371/journal.pone.0275151)
Supplement: Attachment — Submitted filename: Responses to Reviewers_July 30_2022.docx [file pone.0275151.s007.docx]

**Review Comments to the Author**

Reviewer #1: The authors of this study investigated the epidemiology of some of the key public health issues in Bangladesh, a country with major public health issues and challenges. Although the communicable and infectious diseases have been the main health issue in this country in the past decades, the urbanizing and aging population of country have led to increase in prevalence and incidence of non-communicable and chronic diseases and conditions in the past years. This study benefits a rigor data and analysis; however, the manuscript could benefit from some improvements. My comments and suggestions are provided below in this regard to help making any decision on this submission.
1. Title: the body of manuscript shows that this study evaluates a couple of conditions; however, the title is not inclusive of all these conditions and somehow is misleading. For example, using the term “non-communicable diseases” when only diabetes and hypertension is investigated in study, is confusing and inappropriate. It is suggested that authors kindly revise the title of manuscript.

Response: We have replaced “non-communicable diseases” to “diabetes and hypertension” in the title as suggested.

2. General: language and grammar edit are necessary for the manuscript to enhance its readability.

Response: We have edited language and grammar of the manuscript as suggested.

3. Abstract: the provided results in abstract are too much making following the results hard. It is suggested that authors provide only main results here and keep the rest for the main text of manuscript.

Response: We have edited the manuscript to make it more concise (lines 35-39).

4. Introduction: adding some information on the recent statistics of included conditions using updated references like the recent Global Burden of Diseases (GBD) 2019 estimations, for Bangladesh, could provide an interesting context in this section.

Response: We have added some information on recent statistics from the Global Burden of Diseases 2019 estimations for Bangladesh in lines 65-68.

5. Methods, Determinants of malnutrition and NCDs, lines 135-147: the first part of this section seems to be irrelevant to what should be provided in this section. The provided material could be moved to introduction or discussion sections.

Response: We have shortened that section by removing the 1^st^ paragraph (lines 140-152) and cited the references (line 154, reference 39-41) for the choice of determinants of malnutrition and NCDs.

6. Methods: the details of the regression decomposition analysis is missing and a comprehensive expansion on this method is necessary in the corresponding part of the methods section.

Response: We have added details of the regression decomposition analysis in lines 190-195.

7. Results, tables, and figures: well prepared.

Response: Thank you for the encouraging comment

8. Discussion: alongside the other sections of the manuscript, although this investigation explores numerous conditions and related factors and variables, the one major point which needs to be focused through the text and specifically in the discussion section is missing. The authors may choose one as the major issue and finding of this study and expand the discussion a little more ton the chosen challenge.

Response: This paper and the discussion is necessarily broad given the multiple forms of malnutrition covered. We feel that it is important to consider these conditions together as they do not exist in isolation and interventions must not address one while making another worse. Examining both trends in multiple forms of malnutrition over time and how these forms have changed by population subgroup (e.g. rich and poor) is important for program targeting. Our preference is not to focus on one point but to present the full picture of these conditions and discuss our findings in a holistic manner. We explain our findings by putting them into the economic, dietary, and programmatic contexts (each of these is covered in one paragraph) of Bangladesh.

Reviewer #2: This study aims to explore BMI, Diabetes, and hypertension status in Bangladesh. The dataset is carefully selected for this purpose, and the chosen aims are real public health concerns. For this purpose, statistical methods and data presentation techniques (Figures) were chosen appropriately. The manuscript was written adequately in terms of introduction and methods. However, the results and discussion were not satisfactory. The reason could be establishing too many aims for a single study.

Therefore, I prefer to share my critical concerns:
The aims of the study in lines 81:84 are relatively generally stated. In such research, we need to specify objectives. This issue caused other drawbacks to working.

Response: The first aim of the study concisely specifies the outcomes (BMI, underweight, overweight or obesity, diabetes, and hypertension), the time period (2004 to 2018), the study population (women and men in Bangladesh), and the levels of analysis (national and subnational). The second aim regarding inequities uses plain language to specify the types of inequities (wealth and residence) being examined. The third aim was simply to look at what factors contributed to change. We have added some examples of factors in case this was unclear (line 88).

The title does not reflect the work accurately. It contains underweight, overweight/obesity as specific terms but summarizes diabetes and hypertension in con communicable disease terms. In addition, Using "or" state (/) in the title is not attractive.

Response: We have revised the title as your suggestion, with the specific terms “diabetes and hypertension” for non-communicable diseases and replaced (/) by “or” (lines 1-2).

The abbreviation should be mentioned in the first place of the text. For instance, look at lines 28 and 174. There are two different approaches for SII and CIX. In addition, the common abbreviation for concentration index is (CI). In some work, the authors prefer CIX to differentiate between this and the confidence interval. It does not satisfy your assignment.

Response: We have mentioned the abbreviation of slope index of inequality (SII) and concentration index (CIX) in the first place of the text (line 28).

The wealth index changes a lot over the years, as you mentioned in lines 56 to 64. How could you differentiate the decrease in wealth index variation and inequality of outcomes related to WI?

Response: We selected similar variables (including household ownership of different assets, livestock, house and land, and key housing characteristics) between two rounds to construct wealth index – thus we were able to compare how wealth index changes over time. We categorized wealth index within each round into quintile – and compare the relative inequity of outcome in each round.

You should select one of these approaches: 1- explore the trend of outcomes or 2- explore health inequality across time. Following both of these aims leads to non sufficiently discussion and conclusion for both. Depending on your choice, you need to change the initial results with supplementary results or vice versa.

Response: While we understand the concern regarding the breadth of the paper, we respectfully disagree with the comment of choosing one aims only. Having both aims give the full pictures of the trends over time, and how these changes differ by different groups of population. Reporting both trends and inequities is common in the literature and is important for program targeting.

It seems more informative to explore overweight and obesity as the different outcomes.
The BMI category could be a relevant factor in the decomposition of diabetes and hypertension if you focus only on these outcomes.

Response: The table below showed and increased trend in overweight and obesity over time with equal distribution among men and women till 2011. Obesity increased more than overweight among women in 2014 and 2018. Because overweight and obesity shared similar risk factors and mechanism, we kept them as one outcome in the inequity and regression analyses

|  | **2004** | **2007** | **2011** | **2014** | **2018** |
| --- | --- | --- | --- | --- | --- |
| Women |  |  |  |  |  |
| Overweight | 8.4 | 10.0 | 12.6 | 15.4 | 16.8 |
| Obesity | 8.9 | 11.8 | 15.9 | 25.3 | 30.8 |
| Men |  |  |  |  |  |
| Overweight |  |  | 11.4 |  | 15.1 |
| Obesity |  |  | 8.4 |  | 17.5 |

Table 1: Please add percentages or standard deviation

Response: We have included the standard deviation with the mean for continuous variables in Table 1 as suggested.

Reviewer #3: This prevalence study by Nguyen, P. H., et al., 2022 used representative data from five rounds of Bangladesh Demographic and Health Surveys (BDHS) (2004, 2007, 2011, 2014 and 2018) at both national and division levels. The survey used a two-stage stratified sampling strategy, and a sampling frame was obtained from the list of enumeration areas of the Population and Housing Census of the People’s Republic of Bangladesh, provided by the Bangladesh Bureau of Statistics.
Dependent (outcome) variables: underweight, overweight/obesity, hypertension, diabetes
Independent variables: categorized (in both women and men) at household level (size, SES, exposure to media, rural/urban, hygiene and sanitation) and individual level (age, education, employment).

A total of 74,798 households were surveyed across the five rounds, among them 76,667 non-pregnant women aged 15-49y and 10,900 men aged 18-95y having height and weight measurement data. The number of non-pregnant women and men aged 35-95y having data on NCDs were 7,333 and 6,821 respectively. All analyses were conducted using Stata 17 (StataCorp 187 LLC, College Station, TX, USA). Statistical significance was considered at p < 0.05. This study was a secondary data analysis of BDHS data, which was approved by the institutional review board at ICF and the Bangladesh Medical Research Council. All respondents in the BDHS undergo an informed consent process for participation in the survey.

Results revealed that on average across survey rounds, women were 30 years old, men were 42-45 years old and most (72-78%) lived in rural areas. Over time, household size reduced
197 from 5.6 to 5.1 members and wealth index increased from 2.1 to 6.1. Hygiene and sanitation also improved by 22 percentage points (pp). Average years of education increased for women (from 3.4 years in 2004 to 5.6 years in 2018) and men (4.6 years in 2011 to 5.6 years in 2018). Women participating in labor workforce also increased over time.

The authors made all data underlying the findings fully available. The data was tested for representativeness, analyzed using descriptive and inferential statistics which were rigorous and appropriate.

Discussions of the results were robust, citing similar studies conducted both within and outside Bangladesh.

Conclusions are in line with the findings

Writing quality and clarity: Satisfactory

Other observations:
1. Limitations of the study: The authors did well to mention the limitations of the study

Response: Thank you for the positive comment from the reviewer.

2. Inclusion/exclusion criteria not clearly explained as a separate sub topic

Response: We have added a sentence stating the inclusion criteria in lines 109-110.

References: The manuscript employed the use of Harvard style referencing but requires editing to correct some errors noticed e.g., Listing of references: Shouldn’t this be in alphabetical order? Shouldn’t the journal name be italics? Shouldn’t the list of authors that are more than 5 be reflected as et al?
I suggest the authors should revise Harvard referencing style and make necessary corrections.

Response: We have revised the references according to the requirement from PLOS ONE journal. The PLOS ONE suggested that references are listed and numbered in the order that they appear in the text.

Reviewer #4: The authors assessed the trends of non-communicable diseases and factors that have contributed to NCDs in Bangladesh. This study could have a substantial impact on improving the health system in Bangladesh.

I would suggest some points below:

General comment:
Tables and Figures need a detailed description in the text or a detailed caption. For example, Table 1 needs an explanation of the summary statistic based on variables type.
Response: We have revised tables and figures to meet the requirement of the journal.

Methods:
1) page 5, lines 98-99: an explanation of the reason for choosing a subsample of men only, and not including women.

Response: The height and weight were measured for women in all five rounds, thus we analyzed all data for women. However, data for men were only available in 2 rounds. We have clarified that sentence. (lines 103-104).

2) The details of variables can be transferred to the supplementary appendix.

Response: The PLOS ONE journal required us to “provide enough detail to allow suitably skilled investigators to fully replicate the study”. Therefore, we would like to keep details of variable description in the main manuscript. We have shortened that section by removing the 1st paragraph and cited the references for the choice of determinants of malnutrition and NCDs.

3) To address the potential bias related to each data set in the analysis, you need to undertake a sensitivity analysis.

Response: We have compared characteristics of women with and without anthropometric or NCD data and found they have similar characteristics, thus the sample with available data are representative. Please see the table below:

**Support Table. Characteristics of households, women and men by survey round and availability of data, Bangladesh 2004 – 2018**

| **Data for BMI or diabetes/hypertension** | **2004** | | **2007** | | **2011** | | **2014** | | **2018** | |
| --- | --- | --- | --- | --- | --- | --- | --- | --- | --- | --- |
|  | **Available data** | **Missing data** | **Available data** | **Missing data** | **Available data** | **Missing data** | **Available data** | **Missing data** | **Available data** | **Missing data** |
| **Characteristics of sample for BMI analyses** |  |  |  |  |  |  |  |  |  |  |
| **Household** |  |  |  |  |  |  |  |  |  |  |
| Place of residence (rural), % | 77.7 | 67.2 | 77.6 | 76.5 | 75.0 | 76.4 | 72.0 | 62.7 | 72.3 | 76.7 |
| Household size, *n* | 5.6±2.8 | 5.3±1.6 | 5.5±2.7 | 3.4±1.4 | 5.2±2.5 | 5.3±2.3 | 5.1±2.4 | 5.8±2.8 | 5.1±2.5 | 5.9±3.1 |
| SES index (0-10), score (mean ± SD) | 2.1±2.3 | 2.6±2.2 | 4.1±2.7 | 3.1±1.9 | 5.2±2.5 | 5.1±2.6 | 5.7±2.4 | 6.6±3.4 | 6.1±2.1 | 5.8±2.1 |
| Exposed to media, % | 44.9 | 49.5 | 46.7 | 44.5 | 47.2 | 46.4 | 48.1 | 54.6 | 50.2 | 47.9 |
| Improved latrine, % | 60.5 | 54.2 | 41.8 | 42.4 | 54.8 | 55.8 | 70.2 | 70.8 | 64.2 | 67.8 |
| Improved drinking water, % | 95.6 | 100 | 97.3 | 100 | 98.5 | 99.4 | 97.6 | 98.5 | 98.2 | 98.9 |
| **Women 15-49y** |  |  |  |  |  |  |  |  |  |  |
| Age, y (mean ± SD) | 30.0±9.3 | 25.3±8.0 | 30.4±9.3 | 39.4±3.5 | 31.2±9.2 | 38.8±8.4 | 30.8±9.1 | 26.9±10.5 | 31.6±9.2 | 33.3±9.2 |
| Education, y (mean ± SD) | 3.4±3.7 | 4.3±3.0 | 4.0±3.8 | 3.8±2.0 | 4.7±3.9 | 5.0±5.2 | 5.1±4.0 | 5.4±5.5 | 5.6±4.0 | 6.2±5.3 |
| Currently working, % | 21.5 | 17.9 | 32.7 | 27.6 | 14.2 | 12.1 | 33.9 | 39.5 | 49.1 | 40.4 |
| Currently married, % | 100 | 100 | 100 | 100 | 100 | 97.9 | 100 | 100 | 98.2 | 92.5 |
| Having children <5y, % | 58.1 | 54.9 | 54.7 | 57.6 | 51.3 | 49.8 | 49.2 | 41.7 | 47.8 | 43.8 |
| **Men 18-95y** |  |  |  |  |  |  |  |  |  |  |
| Age, y (mean ± SD) | -- | -- | -- | -- | 45.2±15.3 | 48.7±16.7 | -- | -- | 41.6±16.7 | 47.7±22.9 |
| Education, y (mean ± SD) | -- | -- | -- | -- | 4.6±4.5 | 4.2±4.7 | -- | -- | 5.6±4.7 | 5.4±5.0 |
| Manual worker, % | -- | -- | -- | -- | 50.9 | 45.8 | -- | -- | 53.0 | 55.4 |
| Currently married | -- | -- | -- | -- | 99.7 | 99.1 | -- | -- | 83.8 | 88.8 |
| **Characteristics of sample for diabetes and hypertension analyses** |  |  |  |  |  |  |  |  |  |  |
| **Household** |  |  |  |  |  |  |  |  |  |  |
| Place of residence (rural) , % | -- | -- | -- | -- | 75.8 | 74.1 | -- | -- | 74.0 | 73.0 |
| Household size, *n* (mean ± SD) | -- | -- | -- | -- | 5.1±2.4 | 5.0±2.3 | -- | -- | 4.9±2.3 | 4.8±2. |
| SES index (0-10), score (mean ± SD) | -- | -- | -- | -- | 5.2±2.6 | 5.2±2.5 | -- | -- | 6.0±2.2 | 6.1±2.1 |
| Exposed to media, % | -- | -- | -- | -- | 47.2 | 47.3 | -- | -- | 49.2 | 50.8 |
| Improved latrine, % | -- | -- | -- | -- | 55.4 | 54.3 | -- | -- | 64.7 | 65.6 |
| Improved drinking water, % | -- |  | -- |  | 98.4 | 98.6 | -- | -- | 98.0 | 98.0 |
| **Women 35-95y** |  |  |  |  |  |  |  |  |  |  |
| Age, y (mean ± SD) | -- | -- | -- | -- | 45.8±10.3 | 41.5±4.3 | -- | -- | 45.1±9.5 | 41.6±4.4 |
| Education, y (mean ± SD) | -- | -- | -- | -- | 2.7±3.7 | 3.0±3.9 | -- | -- | 3.3±3.9 | 3.6±3.9 |
| Currently working, % | -- | -- | -- | -- | 12.9 | 14.8 | -- | -- | 52.3 | 57.2 |
| Currently married, % | -- | -- | -- | -- | 99.9 | 100 | -- | -- | 99.9 | 100 |
| Having children <5y, % | -- | -- | -- | -- | 31.6 | 28.2 | -- | -- | 29.7 | 26.1 |
| **Men 35-95y** |  |  |  |  |  |  |  |  |  |  |
| Age, y (mean ± SD) | -- | -- | -- | -- | 51.9±12.8 | 52.3±9.2 | -- | -- | 51.5±13.2 | 48.6±8.5 |
| Education, y (mean ± SD) | -- | -- | -- | -- | 4.4±4.7 | 5.4±4.4 | -- | -- | 4.6±4.7 | 4.4±3.2 |
| Manual worker, % | -- | -- | -- | -- | 50.9 | 42.4 | -- | -- | 55.4 | 49.6 |
| Currently married, % | -- | -- | -- | -- | 99.6 | 100 | -- | -- | 99.5 | 100 |

4) In table 2, the number and percentage of individuals with the outcome need to be added as separate columns for each NCD's risk factor.

Response: We have added a separate table of the number and percentage of individuals with the outcome in S4 Table. We prefer not adding more column or statistics in Table 2- because it would make Table 2 too crowded.

Journal Requirements:

When submitting your revision, we need you to address these additional requirements.

1. Please ensure that your manuscript meets PLOS ONE's style requirements, including those for file naming. The PLOS ONE style templates can be found at 
[https://journals.plos.org/plosone/s/file?id=wjVg/PLOSOne_formatting_sample_main_body.pdf](https://eur03.safelinks.protection.outlook.com/?url=https%3A%2F%2Fjournals.plos.org%2Fplosone%2Fs%2Ffile%3Fid%3DwjVg%2FPLOSOne_formatting_sample_main_body.pdf&data=05%7C01%7Cp.h.nguyen%40cgiar.org%7C7827f6b53689452fc83108da6b4a5c52%7C6afa0e00fa1440b78a2e22a7f8c357d5%7C0%7C0%7C637940264957162707%7CUnknown%7CTWFpbGZsb3d8eyJWIjoiMC4wLjAwMDAiLCJQIjoiV2luMzIiLCJBTiI6Ik1haWwiLCJXVCI6Mn0%3D%7C3000%7C%7C%7C&sdata=HvIH7wfFVS%2BbIYWIV7w1JcHimHCpnk9OvilYru9Crf4%3D&reserved=0) and 
[https://journals.plos.org/plosone/s/file?id=ba62/PLOSOne_formatting_sample_title_authors_affiliations.pdf](https://eur03.safelinks.protection.outlook.com/?url=https%3A%2F%2Fjournals.plos.org%2Fplosone%2Fs%2Ffile%3Fid%3Dba62%2FPLOSOne_formatting_sample_title_authors_affiliations.pdf&data=05%7C01%7Cp.h.nguyen%40cgiar.org%7C7827f6b53689452fc83108da6b4a5c52%7C6afa0e00fa1440b78a2e22a7f8c357d5%7C0%7C0%7C637940264957162707%7CUnknown%7CTWFpbGZsb3d8eyJWIjoiMC4wLjAwMDAiLCJQIjoiV2luMzIiLCJBTiI6Ik1haWwiLCJXVCI6Mn0%3D%7C3000%7C%7C%7C&sdata=ZIK50Q6%2Fq1G2cAjpOQxlHZGGbMfxt%2Fee2jzEnBUVfmE%3D&reserved=0)

Response: We have checked and ensured that our revised manuscript meet PLOS ONE's style requirements

2. Please provide additional details regarding participant consent. In the ethics statement in the Methods and online submission information, please ensure that you have specified what type you obtained (for instance, written or verbal, and if verbal, how it was documented and witnessed). If your study included minors, state whether you obtained consent from parents or guardians. If the need for consent was waived by the ethics committee, please include this information.

Once you have amended this/these statement(s) in the Methods section of the manuscript, please add the same text to the “Ethics Statement” field of the submission form (via “Edit Submission”).

For additional information about PLOS ONE ethical requirements for human subjects research, please refer to [http://journals.plos.org/plosone/s/submission-guidelines#loc-human-subjects-research](https://eur03.safelinks.protection.outlook.com/?url=http%3A%2F%2Fjournals.plos.org%2Fplosone%2Fs%2Fsubmission-guidelines%23loc-human-subjects-research&data=05%7C01%7Cp.h.nguyen%40cgiar.org%7C7827f6b53689452fc83108da6b4a5c52%7C6afa0e00fa1440b78a2e22a7f8c357d5%7C0%7C0%7C637940264957162707%7CUnknown%7CTWFpbGZsb3d8eyJWIjoiMC4wLjAwMDAiLCJQIjoiV2luMzIiLCJBTiI6Ik1haWwiLCJXVCI6Mn0%3D%7C3000%7C%7C%7C&sdata=tSjwF6yWft9rytUh6VYrZ7D52T5loKNciFoyuH8N9M8%3D&reserved=0).

Response: We have provided additional details regarding participant consent in Ethics Statement in the Method section of the manuscript (lines 204-207), and also add in the submission form.

“The enumerators read a detailed informed consent statement to the respondents, informing about the survey, describing the procedure, and emphasizing the voluntary nature of participation. Respondents will sign the consent form once they agreed to participate in the survey”.

3. We note that the grant information you provided in the ‘Funding Information’ and ‘Financial Disclosure’ sections do not match.

When you resubmit, please ensure that you provide the correct grant numbers for the awards you received for your study in the ‘Funding Information’ section.

Response: we have now provided matching information of the grant in the ‘Funding Information’ and ‘Financial Disclosure’ sections.

4. Thank you for stating the following in the Acknowledgments Section of your manuscript:

"Funding for this research was provided by the Bill & Melinda Gates Foundation through Alive & Thrive, managed by FHI Solutions (grant numbers: OPP1170427) and the CGIAR Research Program on Agriculture for Nutrition and Health (A4NH), led by the International Food Policy Research Institute (IFPRI)."

We note that you have provided funding information that is not currently declared in your Funding Statement. However, funding information should not appear in the Acknowledgments section or other areas of your manuscript. We will only publish funding information present in the Funding Statement section of the online submission form. 
Please remove any funding-related text from the manuscript and let us know how you would like to update your Funding Statement. Currently, your Funding Statement reads as follows:

"Funding for this research was provided by the Gates Foundation through Alive & Thrive, managed by FHI Solutions (grant numbers: OPP1170427). The funders had no role in study design, data collection and analysis, data interpretation, decision to publish, or preparation of the manuscript."

Please include your amended statements within your cover letter; we will change the online submission form on your behalf.

Response: We have removed funding-related text from the manuscript (lines 425-429) and updated our funding statement as follow:

“"Funding for this research was provided by the Bill & Melinda Gates Foundation through Alive & Thrive, managed by FHI Solutions (grant numbers: OPP1170427) and the CGIAR Research Program on Agriculture for Nutrition and Health (A4NH), led by the International Food Policy Research Institute (IFPRI). The funders had no role in study design, data collection and analysis, data interpretation, decision to publish, or preparation of the manuscript."

5. We note that Figures 3 and 4 in your submission contain [map/satellite] images which may be copyrighted. All PLOS content is published under the Creative Commons Attribution License (CC BY 4.0), which means that the manuscript, images, and Supporting Information files will be freely available online, and any third party is permitted to access, download, copy, distribute, and use these materials in any way, even commercially, with proper attribution. For these reasons, we cannot publish previously copyrighted maps or satellite images created using proprietary data, such as Google software (Google Maps, Street View, and Earth). For more information, see our copyright guidelines: [http://journals.plos.org/plosone/s/licenses-and-copyright](https://eur03.safelinks.protection.outlook.com/?url=http%3A%2F%2Fjournals.plos.org%2Fplosone%2Fs%2Flicenses-and-copyright&data=05%7C01%7Cp.h.nguyen%40cgiar.org%7C7827f6b53689452fc83108da6b4a5c52%7C6afa0e00fa1440b78a2e22a7f8c357d5%7C0%7C0%7C637940264957162707%7CUnknown%7CTWFpbGZsb3d8eyJWIjoiMC4wLjAwMDAiLCJQIjoiV2luMzIiLCJBTiI6Ik1haWwiLCJXVCI6Mn0%3D%7C3000%7C%7C%7C&sdata=3Xi2YbwKS22ISd0HI0R9YEjaox1hAoZajs1onwofkBE%3D&reserved=0).
 
We require you to either (1) present written permission from the copyright holder to publish these figures specifically under the CC BY 4.0 license, or (2) remove the figures from your submission:
 
a. You may seek permission from the original copyright holder of Figures 3 and 4 to publish the content specifically under the CC BY 4.0 license.  
 
We recommend that you contact the original copyright holder with the Content Permission Form ([http://journals.plos.org/plosone/s/file?id=7c09/content-permission-form.pdf](https://eur03.safelinks.protection.outlook.com/?url=http%3A%2F%2Fjournals.plos.org%2Fplosone%2Fs%2Ffile%3Fid%3D7c09%2Fcontent-permission-form.pdf&data=05%7C01%7Cp.h.nguyen%40cgiar.org%7C7827f6b53689452fc83108da6b4a5c52%7C6afa0e00fa1440b78a2e22a7f8c357d5%7C0%7C0%7C637940264957162707%7CUnknown%7CTWFpbGZsb3d8eyJWIjoiMC4wLjAwMDAiLCJQIjoiV2luMzIiLCJBTiI6Ik1haWwiLCJXVCI6Mn0%3D%7C3000%7C%7C%7C&sdata=NCtD2fdAJI8rVbZIqqLgPHFknW2esB7mIWAUZHas9Eo%3D&reserved=0)) and the following text:
“I request permission for the open-access journal PLOS ONE to publish XXX under the Creative Commons Attribution License (CCAL) CC BY 4.0 ([http://creativecommons.org/licenses/by/4.0/](https://eur03.safelinks.protection.outlook.com/?url=http%3A%2F%2Fcreativecommons.org%2Flicenses%2Fby%2F4.0%2F&data=05%7C01%7Cp.h.nguyen%40cgiar.org%7C7827f6b53689452fc83108da6b4a5c52%7C6afa0e00fa1440b78a2e22a7f8c357d5%7C0%7C0%7C637940264957162707%7CUnknown%7CTWFpbGZsb3d8eyJWIjoiMC4wLjAwMDAiLCJQIjoiV2luMzIiLCJBTiI6Ik1haWwiLCJXVCI6Mn0%3D%7C3000%7C%7C%7C&sdata=svfzsO6DcMnjlfOZLgGRFFnOWiO4apj9539Cum0agHo%3D&reserved=0)). Please be aware that this license allows unrestricted use and distribution, even commercially, by third parties. Please reply and provide explicit written permission to publish XXX under a CC BY license and complete the attached form.”
 
Please upload the completed Content Permission Form or other proof of granted permissions as an ""Other"" file with your submission. In the figure caption of the copyrighted figure, please include the following text: “Reprinted from [ref] under a CC BY license, with permission from [name of publisher], original copyright [original copyright year].”
 
b. If you are unable to obtain permission from the original copyright holder to publish these figures under the CC BY 4.0 license or if the copyright holder’s requirements are incompatible with the CC BY 4.0 license, please either i) remove the figure or ii) supply a replacement figure that complies with the CC BY 4.0 license. Please check copyright information on all replacement figures and update the figure caption with source information. If applicable, please specify in the figure caption text when a figure is similar but not identical to the original image and is therefore for illustrative purposes only.
Response: We created the figures ourselves using a shapefile which is an empty map that is publicly available. The shape file was taken from Global admin boundaries (GADM) <http://gadm.org> and do not violate copyright issues. We have added the note under Figures 3 and 4.

“Note: This map is created from the shapefile (an empty map) that is publicly available from <http://gadm.org>”.
