# Supplementary material for: Underweight, overweight or obesity, diabetes, and hypertension in Bangladesh, 2004 to 2018
Source: PLoS One. 2022 Sep 30;17(9):e0275151. doi: 10.1371/journal.pone.0275151 (PMC9524627; doi:10.1371/journal.pone.0275151)
Supplement: Attachment — Submitted filename: Responses to Reviewers_Sep 10_2022.docx [file pone.0275151.s008.docx]

**Responses to Reviewers**

Thank you very much for the critical comments and suggestions. We have completed the revisions following your recommendation in details as below

**Reviewer #1:** The authors addressed the comments adequately and provided reasonable responses in this revision and the response letter, regarding my comments. The manuscript is acceptable in this format in my opinion.

Response: Thank you for your additional review and approved our revision.

**Reviewer #2**: The authors answered my concerns carefully. I want to thank them. However, there are several concerns:

1. It is arbitrary in Plos one style to write the abstract in a structured format. However, adding a title for each part make the abstract more readable.

Response: Thank you for your comment. As suggested, we have formatted our abstract to be a structured abstract.

2. The age period cohort effect is demonstrated in figure 1. It would help if you discussed this effect more.

Response: we have commented on the age cohort effects on BMI in line 208-211. We now added S1 Figure and discussed on age cohort effects on underweight and overweight/obesity (lines 211-213).

3. The higher degree of smoothness in statistical models could be misleading. Adding observed points to the smooth lines in Figures 1 and 2 could be informative.

Response: We agree with the Reviewer that adding the observed points to the smooth lines in Figure 1 and 2 could give more information. However, given the data are large (76,758 data point for women and 10,494 data points for men), adding observed points in this type of graph causes overplotting, making the graphs noisy. Therefore, we decided to keep the graphs as original to deliver a clearer message.

**Reviewer #3:** All my previous comments have been adequately addressed. The paper is now Ok and acceptable for publication.

Response: Thank you for your additional review and recommended our manuscript for publication.

**Reviewer #4**: The authors perfectly addressed the comments, which improved the results and methodology

1. I would suggest including the percentage [number(and %)] of individuals at each study round contributed to the whole analysis. For example, the number of individuals in 2004 and the percentage of the data for this year compared to the total number of individuals included in the analysis. It helps to have an overview of the study size in one look at the table

Response: Thank you for the comment. As your suggestion, we have added the proportion of sample from specific round to the total sample size in the Table 1.
